# Health care users’ acceptance of broad consent for storage of biological materials and associated data for research purposes in Uganda

**DOI:** 10.12688/wellcomeopenres.17633.2

**Published:** 2022-10-04

**Authors:** Hellen Nansumba, Mugalula Flaviano, Semanda Patrick, Ssewanyana Isaac, Douglas Wassenaar

**Affiliations:** 1 Central Public Health Laboratories (CPHL), Ministry of Health of Uganda, Kampala, Uganda; 2 South African Research Ethics Training Initiative (SARETI), University of KwaZulu-Natal, South Africa, Kwazulu-Natal, South Africa

**Keywords:** Broad consent, biobanking, comprehension, informed consent form, biological materials

## Abstract

**Background:** Implementation of appropriate informed consent has become a cornerstone for the use of biological materials and data from clinical care to use in research. During 2017–2018, the Ugandan National Biorepository has since sought prior informed consent for long-term storage and use of remnant clinical human biological materials, where a shortened informed consent form (ICF) was incorporated on the laboratory investigation form. This project aimed at determining the acceptability rate of broad consent from health care users (HCUs) for storage of biological materials and data for research purposes in Uganda.

**Methods:** A cross-sectional study was conducted at three Primary Health Care Facilities. 500 HCUs above 18 years of age seeking health care at outpatient departments between March to December 2020 were invited to enrol. A shortened experimental ICF for this study was developed and attached to the Laboratory investigation form.

**Results:** Overall the acceptability of broad consent for storage of biological materials and data was 86.2% [95% CI: 82.9%-88.9%]. Compared to participants who perceived that the informed consent information is understandable (OR=0.10, CI [0.03-0.32], participants who either partly or totally disagreed were significantly less likely to perceive information as understandable (OR=0.27, CI [0.15-0.46]. 226 out of 431 respondents that accepted storage of biological materials and data, majority (61.7%) preferred to receive feedback on results of relevance to their health.

**Conclusion:** Acceptance of broad consent for storage of biological materials and data for future research purposes was high among HCUs. A shortened and simplified ICF may trigger discussions between participants and health care workers hence increase research participant understanding of study related materials in biobanking. This in turn could enrich ethically collected biobank resources for future research of public health relevance.

## Background

Uganda adopted a centralized model of testing to scale-up its national Health programmes such as HIV Early Infant Diagnosis (EID) and HIV viral load monitoring (VL) programmes. Human biological materials such as dried blood spots (DBS) and plasma are collected from remote health facilities in Uganda and delivered to HUBS. A HUB is a coordination center of the sub-district network serving approximately 20–40 health facilities where several referral tests are done. Currently, there are 100 functional HUBS bringing together a network of over 2,500 heath facilities. Biological materials are transported from the HUB to the Central Public Health Laboratory for testing ^1^. The total national coverage of both EID and VL for over 150,000 HIV exposed infants and 1,200,000 HIV patients on Antiretroviral Therapy (ART) has resulted in the collection of over 1,500,000 remnant biological materials in a Biorepository for future research.

In September 2016, a National Biorepository was proposed and established to enable storage of human biological materials in a retrievable manner for future research purposes and to foster both local and international research collaborations. During 2017–2018, the National Biorepository sought prior informed consent for long-term storage and use of remnant clinical human biological materials mainly from the programmes mentioned above. A shortened informed consent form has been incorporated on the laboratory request form ^2^.

Several bottlenecks in governance and regulation of Biobanks in LMICs were highlighted in our previous work ^3^. National Research Biobanking Guidelines have since been developed. They state that all biological materials and associated data obtained during research, clinical care, public health interventions and surveillance require evidence of documented informed consent from the sample donor or their representative for storage ^4– 6^. Each biospecimen should have documentation of informed consent status ^7^. Consent for Biobanking for specified or unspecified future research raises unique challenges and concerns for informed decision making especially in the context of clinical care. Participants often do not understand much of the information they are provided with and this is attributed to overly complex Informed consent forms ^8^ In most cases, there are competing clinical demands that limit time for HCUs engagement with the informed consent document ^9^. Broad consent provides participants with a choice about whether to allow their stored specimens to be used in future research based on a broad category, e.g., cancer, heart disease, or behavioral research ^10^. However, in the genomics era, broad consent should be implemented within health systems that are trust worthy and have measures in place to ensure that open-ended agreements are not used ^11^. This is because broad consent may provide participants with insufficient information to make a reasonable choice ^12^ while others may change their minds in the future ^13^. Additionally, several studies show the lack of understanding of consent and preferences about broad consent ^14^. Few studies have investigated the African population, so this research conducted is very relevant and needed. 

Implementation of appropriate informed consent is fundamental for the collection and use of biological materials and associated data from clinical care ^4, 15^. This project aimed to understand the acceptance for broad consent and HCUs knowledge, attitudes, and perceptions of broad consent for storage of biological materials and associated data in Uganda. Findings from this study may contribute to current debate regarding understanding the content of informed consent documents, the purpose of biobanks generally, or the scope of the future research and projects to be carried out. Additionally, findings from this study may be used to design evidence-based practices and procedures for obtaining informed consent for biobanking and to inform research ethics committees (RECs) on assessment of biobanking protocols based on the preferences of actual donors ^16, 17^.

## Objectives

**Objective 1:** Determine factors associated with the relationship between understanding of essential elements of the informed consent form and acceptability of storage of biological material and data

**Objective 2:** Determine the factors associated with attitudes and acceptability of storage of biological material and data

**Objective 3:** Determine HCUs acceptability rate for storage of biological materials and associated data

**Objective 4:** Determine factors associated with motivation and acceptability for storage of biological materials and data

**Objective 5:** To determine the factors associated between HCUs’ perceptions and acceptability of storage of biological materials and data

## Methods

The Uganda National Health Laboratory Services Research Ethics Committee (UNHLSREC) for ethics reviewed and approved this study (UNHLSREC008032020). The study was approved by Uganda National Council for Science and Technology (HS652ES). Permission was sought from the Medical officer in-charge of each health facility.

A cross-sectional study design was conducted at three Primary Health Care Facilities; Kiruddu National Referral Hospital, Mukono Health Center IV and Reach-Out Mbuya Community Health Initiative. Two data collection tools were used in this study; (i) a shortened experimental informed consent form and (ii) a semi-structured questionnaire that contained the predictor variables. It is comprised of both closed- and open-ended questions. Both the shortened experimental informed consent form and the questionnaire can be found in the Extended data.

HCUs above 18 years of age seeking health care at outpatient departments (OPDs) between March to December 2020 from the selected Health Facilities (HFs) in Uganda were invited to enrol. Only HCUs who consented were enrolled in this study. HCUs that are were not fluent in English were excluded since the shortened Experimental informed consent form about biobanking was in English language only.

The sample size was estimated using the modified Kish Leslie (1965) formula N= Z ^2^*P (1-P)*D/d ^2^ where: Z= Standard normal value corresponding to the 95% confidence interval= 1.96, P= Meta-analytic results of studies examining comprehension of ‘generic’ domains of informed consent. The proportion of participants that understood the right of withdrawal ^7^ = 0.567, D= Design effect = 1. Because sampling will be implemented in same level of Health facilities, d=Error that can be tolerated in the study=0.05, N= [1.96 ^2^*0.72(1-0.567) *1]/0.05 ^2^ ≈ 479. Thus, a sample size of 500 participants was enrolled in this project.

HCUs at the selected health facilities were approached by trained research assistants and asked whether they were willing to participate. If willing, they were asked to read and sign a study consent form that explained the essential elements of this study to them before they decided whether to participate in this study or not. Once they consented they were asked to study the experimental Informed consent form about biobanking and then complete a closed-ended and open-ended questionnaire on knowledge, perceptions and attitudes towards biobanking.

A shortened experimental informed consent form (ICF) was developed for this study. This was read, understood and “completed” by the participant before the questionnaire was administered. The experimental ICF contained all the essential elements of informed consent such as background to the National Biorepository, purpose of the consent form, examples of possible future research, benefits, risks, costs, refusal to store left-over specimens, return of research findings, inquiry and consent statement. Completion of the experimental ICF was ‘hypothetical’ and agreement with it did not mean that the participants’ samples were actually to be stored in the National Biorepository. It was simply to collect data about the acceptability of HCUs to store human biological material and associated data. A semi-structured questionnaire consisted of both closed- and open-ended questions.

Predictor variables included; Socio-demographic variables (Age, Sex, Level of Education), understanding of informed consent form, attitude towards the informed consent form, motivation of HCUs for storage of biological material and associated data and perceptions of broad consent for storage of biological material and data. Outcome variable was defined as hypothetical acceptability of the HCUs to store biological material and associated data in the National Biorepository.

Outcome variables were computed and presented as percentages with a 95% Confidence Interval. Continuous variables were computed and presented as means and standard deviations while categorical variables were computed and presented as proportions. Independent/ predictor variables were computed and analyzed as percentages with a 95% Confidence Interval. The statistical associations between socio-demographic characteristics, understanding, attitudes, motivation and perception and acceptability to store biological material to the National Biorepository were determined using logistic regression. Odds ratios (OR) and 95% confidence intervals were generated to measure the strength of the association of each factor. All statistical analysis was performed using STATA 14.2.

## Results

### Background characteristics of the HCUs enrolled at selected Primary Health Care Facilities

The mean age of the HCUs was 30.18 years. The proportion of female HCUs was higher (53.80%) than males. The highest percentage (49.40%) of HCUs had attained secondary education. The highest proportion (92.40%) of respondents had never participated in health-related research. Most HCUs (91.20%) did not work in a health-related field.

The lowest proportion of respondents (6.45%) understood that remnant biospecimens are sometimes stored for beyond clinical use. Other background characteristics of participating HCUs are summarized in Table 1.

**Table 1.** Background characteristics of 500 HCUs at selected health facilities in Uganda, 2020.

Characteristics	Health Care Users (n=500)
**Mean Age (SD)**	30.18 (± 9.87)
**Gender *n* (%)**	
Male	231 (46.20)
Female	269 (53.80)
**Level of education *n* (%)**	
Primary	91 (18.20)
Secondary	247 (49.40)
Diploma	61 (12.20)
Tertiary	39 (7.80)
Postgraduate	38 (7.60)
Other *	24 (4.80)
**History of participation in health related research *n* (%)**	
Yes	38 (7.60)
No	462 (92.40)
**History of professional work in a medical field *n* (%)**	
Yes	44 (7.60)
No	456 (91.20)
**Knowledge of what happens to remnant biospecimen** ** after laboratory tests *n* (%)**	
Destroyed immediately	230 (46.37)
Destroyed after a duration of time	64 (12.90)
Stored for a duration of time	32 (6.45)
Stored for research purposes	94 (15.32)
Not sure	76 (15.32)

* Certificate

### Prevalence of acceptability of storage of biological material and data related to the background characteristics of health care users

The overall prevalence of acceptability of storage of biological materials and data among HCUs was high (86.2%, 95% CI [82.9%-88.9%]). Acceptability was higher among the young adults aged 18–35 years (76.6%, 95% CI [72.3%-80.3%]). The prevalence of acceptability was higher among respondents that completed at least secondary education (50.1%, 95% CI [45.4%-54.8%]). Acceptability of storage of biological materials and data for research purposes among HCUs stratified by various background characteristics is summarized in Table 2.

**Table 2.** Prevalence of acceptability of storage of biological material and data related to the background characteristics of health care users in Uganda, 2020.

Characteristics	Frequency	Percent	[95% CI]	p-value
**Overall prevalence**	431	86.2	[82.9-88.9]	--
**Health care users age**				
18–35 years	330	76.6	[72.3-80.3]	N/A
36–55 years	87	20.2	[16.6-24.3]	--
Above 55 years	14	3.3	[1.9-5.4]	--
**Gender**				
Male	192	44.6	[39.9-49.3]	0.069
Female	239	55.5	[50.7-60.1]	--
**Level of education **				
Primary	72	16.7	[13.5-20.5]	N/A
Secondary	216	50.1	[45.4-54.8]	--
Diploma	51	11.8	[9.1-15.3]	--
Tertiary	36	8.4	[6.1-11.4]	--
Postgraduate	34	7.9	[5.7-10.9]	--
Other *	22	5.1	[3.4-7.6]	
**History of participation in health related research**				
Yes	38	8.8	[6.5-11.9]	0.019
No	393	91.2	[88.1-93.5]	--
**History of professional work in a medical field **				
Yes	43	10	[7.5-13.2]	0.005
No	38.8	90	[86.8-92.5]	--
**Knowledge of what happens to remnant** ** biospecimen after laboratory tests**				
Destroyed immediately	187	43.8	[39.1-48.6]	N/A
Destroyed after a duration of time	50	11.7	[8.9-15.1]	--
Stored for a duration of time	28	6.6	[4.6-93.4]	--
Stored for research purposes	91	21.3	[17.7-25.5]	--
Not sure	71	16.3	[13.4-20.5]	--

*Certificate

### Perception of the format of the experimental informed consent form

**Figure 1.**
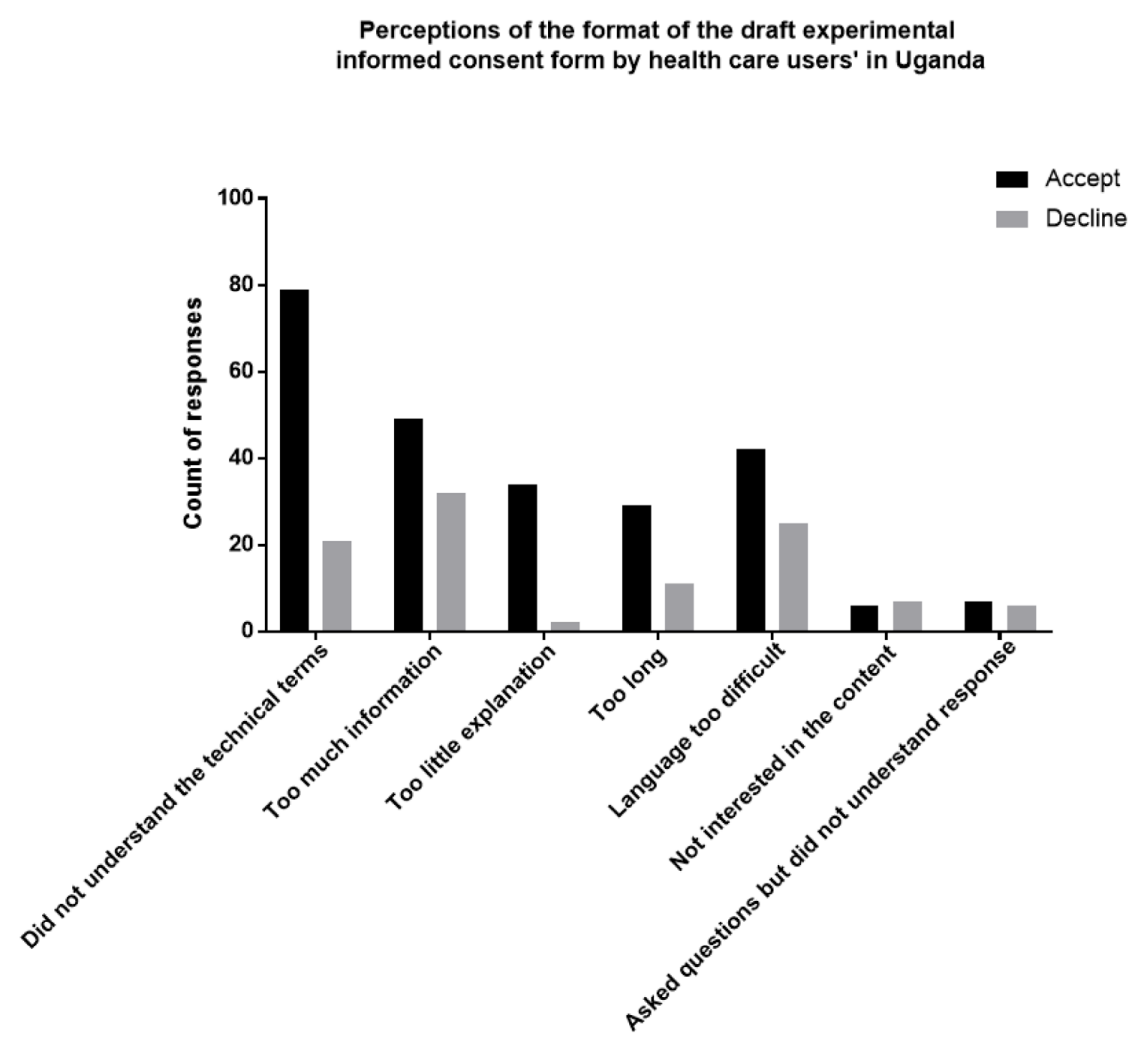
Perception of the format of the experimental informed consent form by HCUs in Uganda, 2020.

Informed consent information that is either partly or not understandable was found to be statistically significant (OR=0.27, 95% CI [0.15-0.46]) and (OR=0.10, 95% CI [0.03-0.32]) respectively as compared to informed consent information perceived as understandable. Less informed consent form information was found to be statistically significant (OR=4.39, 95% CI [2.03-9.53] with acceptability for storage of biological materials and data. Difficult language was found to be statistically significant (OR= 2.65, 95% CI [1.30-5.39]). Length of informed consent form was found not to be statistically significant. HCUs opinion on the format of the informed consent form is summarised in Table 3 and Figure 1.

**Table 3.** Perception of the format of the experimental informed consent form by HCUs in Uganda, 2020.

Variable	‘ *Hypothetica*l’ broad consent for biospecimen storage			
	Accept *n* (%)	Decline *n* (%)	OR [95% CI]	p-value
**Length of informed consent form**				
Too short	17 (3.94)	5 (7.25)	1.0	
Too long	59 (13.72)	19 (27.54)	0.91 [0.30-2.81]	0.874
Just right	354 (82.33)	45 (65.22)	2.31 [0.81-6.57]	0.115
**Information is understandable**				
Yes	307 (71.40)	26 (37.68)	1.0	
No	7 (1.63)	6 (8.70)	0.10[0.03-0.32]	<0.001
Partly	116 (26.98)	37 (53.62)	0.27[0.15-0.46]	<0.001
**Format of the informed consent form**				
**Did not understand the technical terms**				
Yes	79 (65.29)	21 (51.22)	1.0	
No	42 (34.71)	20 (48.78)	0.56 [0.27-1.14]	0.111
**Too much information**				
Yes	49 (39.84)	32 (74.42)	1.0	
No	74 (60.16)	11 (25.58)	4.39[2.03-9.53]	<0.001
**Too little explanation**				
Yes	34 (28.10)	2 (4.88)	1.0	
No	87 (71.90)	39 (95.12)	0.13 [0.03-0.57]	0.007
**Too long**				
Yes	29 (23.97)	11 (26.19)	1.0	
No	92 (76.03)	31 (73.81)	1.13 [0.50-2.52]	0.773
**Language too difficult**				
Yes	42 (34.43)	25 (58.14)	1.0	
No	80 (65.57)	18 (41.86)	2.65 [1.30-5.39]	0.007
**Read too quickly**				
Yes	36 (29.51)	--	1.0	
No	86 (70.49)	42 (100)	--	--
**Not interested in the content**				
Yes	6 (5.08)	7 (17.07)	1.0	
No	112 (94.92)	34 (82.93)	3.84 [1.21-12.2]	0.022
**Asked questions but did not understand** ** response**				
Yes	7 (5.79)	6 (14.29)	1.0	
No	114 (94.21)	36 (85.71)	2.71 [0.86-8.60]	0.090

### Understanding of essential elements of the experimental informed consent form

The majority of respondents understood that their specimen and data could also be used for other health research questions (93.2%, 95%CI [90.6%-95.1%]). A high proportion of the respondents understood they could withdraw consent for storage on specimen and data at any time (84.34%, 95% CI [80.9%-87.3%]). Most HCUs incorrectly believed that their specimens and data would only be used by the National Biorepository (46.48%, 95% CI [42.1%-50.9%]), instead of being accessed from there by other researchers. A summary of HCUs understanding of the essential elements of the informed consent form is described in Table 4: Understanding of essential elements of the experimental informed consent form by HCUs in Uganda, 2020

**Table 4.** Understanding of essential elements of the experimental informed consent form by HCUs in Uganda, 2020.

Characteristics	Correct n (%) [95% CI]	Not correct n (%) [95% CI]	Not sure n (%) [95% CI]
My specimen and data can only be used for research questions that relate to my disease	159 (31.9) [28.0-36.2]	293 (58.95) [54.6-63.2]	45 (9.05) [6.8-11.92]
My specimen and data can also be used for any other medical research question	464 (93.2) [90.6-95.1]	14 (2.81) [1.7-4.7]	20 (4.02) [2.6-6.1]
I can withdraw my consent to store my specimen and associated data at any time	420 (84.34) [80.9-87.3]	16 (3.21) [1.9-5.2]	62 (12.45) [9.8-15.7]
If I have consented that my specimen and associated data are stored, I cannot withdraw this consent	36 (7.23) [5.3-9.9]	342 (68.67) [64.4-72.6]	120 (24.10) [20.5-28.1]
My specimen and data will only be used at the National Biorepository	183 (36.82) [32.7-41.2]	231 (46.48) 42.1-50.9	83 (16.70) [13.7-20.3]
My specimen and data might also be shared with interested researchers who work on relevant medical research projects and have the approval of a research ethics committee.	443 (89.13) [86.0-91.6]	18 (3.62) [2.3-5.7]	36 (7.24) [5.3-9.9]
Any unauthorized tracking back of data to me personally is completely impossible	299 (60.16) [55.8-64.4]	27 (5.43) [3.7-7.8]	171 (34.41) [30.3-38.7]
If in the course of the research with my specimen and data something is found out that is relevant to my health, this will be reported to me	468 (93.79) [91.3-95.6]	--	31 (6.21) [4.4-8.7]

### Motivation of HCUs who accepted storage of biological materials and data for research purposes

The majority of the HCUs accepted storage of biological materials and data to help other future patients with the same disease/health problem (84.35%, 95% CI [80.6%-87.5%]). A low proportion of respondents worried that they might be somehow disadvantaged if they gave consent to store biological materials and data for research purposes (5.9%, 95% CI [3.9%-8.6%]). The various reasons that motivate HCUs to accept storage of biological materials and data are summarized in Table 5.

**Table 5.** Motivation of HCUs who accept storage of biological materials and data for unspecified reasons in Uganda, 2020.

Characteristics	YES n (%) [95% CI]	No n (%) [95% CI]
I hope I will personally benefit from the research with my specimen and data	325 (75.93) [74.6-82.4]	103 (24.07) [17.6-25.4]
I would like to support medical research in general	340 (79.44) [75.3-83.0]	88 (20.56) [17.0-24.7]
I would like to help other future patients with the same disease/health problem	361 (84.35) [80.6-87.5]	67 (15.65) [12.5-19.4]
I would like to benefit from the advantages of medical research; therefore, I should also make a contribution to it.	214 (50) [45.3-54.7]	214 (50) [45.3-54.7]
I would like to act as a role model for other patients	167 (39.02) [34.5-43.7]	261 (60.98) [56.3-65.5]
Being a patient myself, I feel connected to future patients and would like to do something for them	184 (42.99) [38.4-47.7]	244 (57.01) [52.3-61.6]
I am interested in medical research and would like to be part of it	208 (48.6) [43.9-53.4]	220 (51.4) [46.6-56.1]
I know other patients who consented and this persuaded me to consent too	91 (21.6) [17.6-25.4]	337 (78.74) [74.6-82.4]
I am grateful to my doctors and give my consent in order to help them with their work	248 (57.94) [53.2-62.6]	180 (42.06) [37.4-46.8]
I worry that I will be disadvantaged or that my treatment will suffer if I do not give my consent.	25 (5.88) [3.9-8.6]	400 (94.12) [91.4-96.0]
I have not thought about the use of my specimen and data, I do not care	75 (17.77) [14.4-21.7]	347 (82.23) [78.3-85.6]

### HCUs’ attitudes towards return of research findings from biological materials and associated data used for research purposes

**Figure 2.**
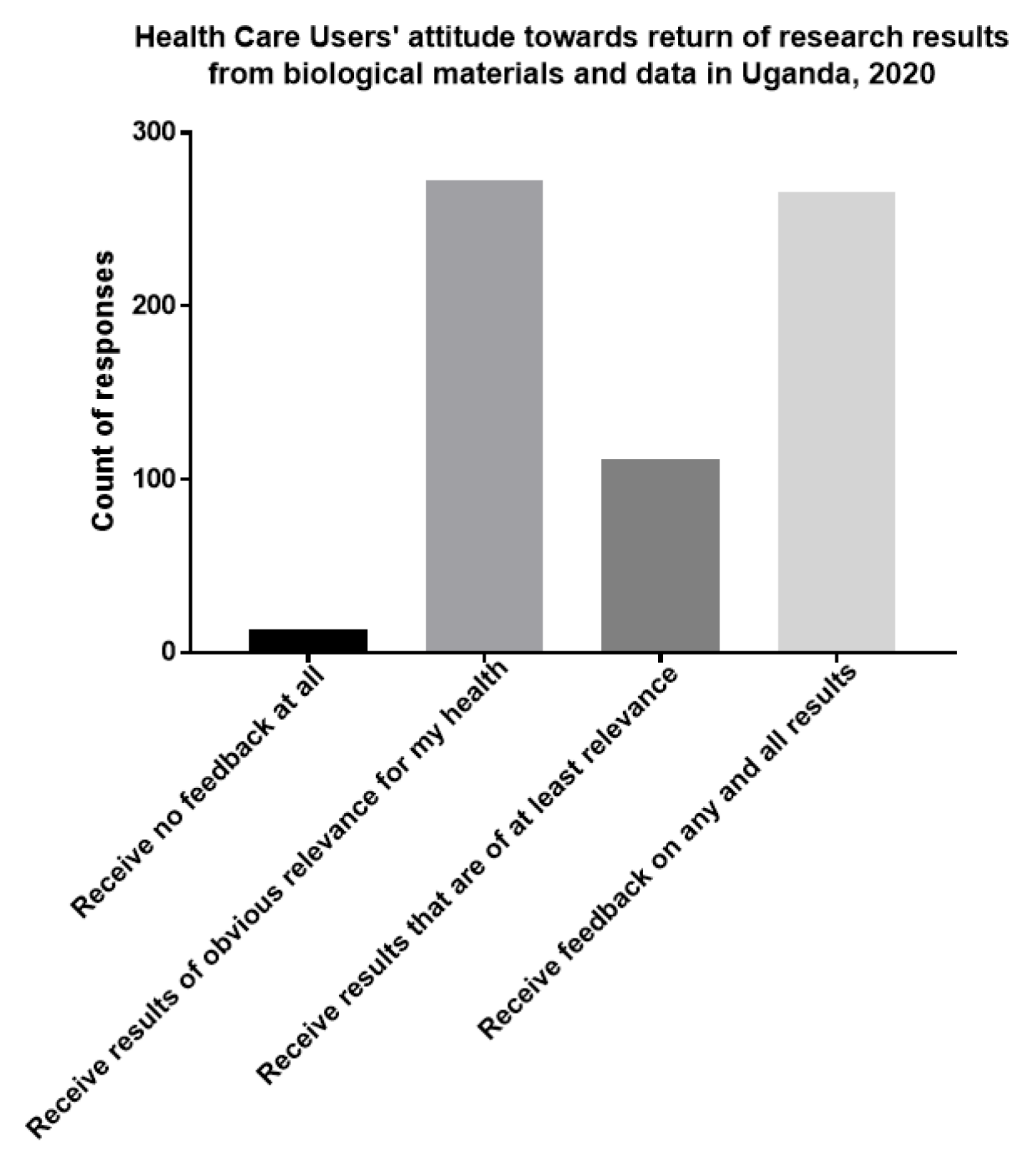
HCUs’ attitudes towards return of research results from biological materials and data in Uganda, 2020.

Among respondents that accepted storage of biological materials and data, the majority (266 of 431) preferred to receive feedback on results of any relevance to their health. Figure 2 summarizes HUCs opinion on the return of research results from utilized biological materials and data.

Reasons given for why HCUs wanted feedback of results of clinical relevance included:

1. Early diagnosis: Understand unexpected medical conditions and access early treatment

2. Understand health complications hence improve health outcomes

3. Understand health status

4. Promote and improves quality of life

5. Improve relevance of research to study populations

6. Research participants are entitled to know all about their health

In summary, acceptability for storage of biological materials and data for research purposes among HCUs was high (86.2%, 95% CI [82.9%-88.9%]). HCUs with a history of participating in health-related research were statistically different from those that had never participated (p-value = 0.019). And HCUs with a history of professional work in a medical field were statistically different from those that worked in non-medical fields (p-value = 0.005).

Compared to participants who perceived that the informed consent information is understandable (OR=0.10, 95% CI [0.03-0.32], participants who either partly or totally disagreed were significantly less likely to perceive information as understandable (OR=0.27, 95% CI [0.15-0.46]. Respondents who perceived the ICF information as brief and concise were four times more likely to accept storage of biological materials and data compared to those who thought it was lengthy (OR=4.39, 95% CI [2.03-9.53]. HCUs who perceived the ICF language as easy to read were 3 times more likely to accept storage of biological materials and data compared to those who found the language quite difficult (OR= 2.65, 95% CI [1.30-5.39]). HCUs opinion on the format of the informed consent form is summarised in Table 3 and Figure 1.

Most respondents understood that their specimen and data could also be used for other health research questions (93.2%, 95% CI [90.6%-95.1%]). A high proportion of respondents understood that they could withdraw consent for storage of specimens and data at any time (84.34%, 95% CI [80.9%-87.3%]). Most HCUs incorrectly believed that their specimens and data would only be used by the National Biorepository (46.48%, 95% CI [42.1%-50.9%]), instead of being accessible from there by other researchers on request. Among respondents that accepted storage of biological materials and data, the majority (266 of 431) preferred to receive feedback on results of relevance to their health. A summary of HCUs understanding of the essential elements of the informed consent form is described in Table 4.

The majority of the HCUs accepted storage of biological materials and data to help other future patients with the same disease/health problem (84.35%, 95% CI [80.6%-87.5%]). A low proportion of respondents worried that they might be somehow disadvantaged if they gave consent to store biological materials and data for research purposes (5.9%, 95% CI [3.9%-8.6%]).

One of the limitations is that the study did not adjust for key confounding variables such as level of education. However, health care users that were illiterate of the English language were excluded from the study since the experimental informed consent form about biobanking was written in English language.

## Discussion

### Prevalence of acceptability for storage of biological materials and data

Acceptance of storage of biological materials and data was found to be high at 86%, suggesting that most participating HCUs *hypothetically* accepted broad consent for storage and use of biological material and data for unspecified and/or broad research purposes. This acceptance was higher than the 49.5% that indicated that they would want to be contacted each time their sample was re-used, as reported by Moodley *et al.* (2014). Additionally, our acceptance rate was higher than the 66.1% that agreed that broad consent was permissible as reported by Mwaka *et al.* (2019).

This could partially be explained by the fact that the concept of broad consent and alternative approaches to consent for re-use of biological materials and data has increasingly been discussed in LMICs ^17– 19^. As mentioned in our Background section ^20, 21^, several challenges have been highlighted in obtaining only specific informed consent; e.g., samples obtained from pediatric and autopsy studies regarded as precious resources, considering the circumstances of the consent process and scarcity of such cases. Biological materials obtained during outbreak investigations may not be traceable to the primary source. Consent to re-use biological materials for unspecified future research helps to lessen the cost and time burdens on researchers.

Almost half of the participants (43.8%) believed that remnant biospecimens were destroyed immediately after laboratory tests. Only 6.6 % and 21.3% thought clinical specimens might be stored for a specified period and for research purposes, respectively. This data may guide in the development of Standard Operating Procedures for sample retention times for biological materials used for clinical purposes that have not been consented for storage. This suggests that sample storage and further use possibilities should be more clearly specified at the time of sampling.

### Perception of format of experimental informed consent form

A shortened experimental Informed consent form was developed and tested with HCUs in a public health setting. One of the objectives of this study was to determine HCUs’ perception of the length and technical content of the informed consent form. Our data suggest that 82.3% of HCUs found that the length of the informed consent form was appropriate. Sixty five percent of HCUs who accepted storage of biological materials and data indicated that they did not understand some technical terms. However, acceptability of storage of biological materials was not a statistically significant preference. Compared to participants who perceived that the informed consent information is understandable, participants who either partly or totally disagreed were significantly less likely to perceive the information as understandable. Lengthy informed consent forms remain common in research settings in LMICs, despite the fact that they negatively affect potential participants’ understanding about the proposed research. A shortened informed consent form may prompt better understanding and willingness to consent, as has been shown elsewhere ^22, 23^.

HCUs responses on how to improve the broad consent form were articulated as follows: (a) Simplify the technical terms and language used. (b) The Informed consent form should be translated in various languages. (c) Shorten the informed consent form to make it brief. (d) Offer participants enough time to understand the information. (e) More sensitization of the informed consent process should be done. (f) The examples for possible re-use of biological materials should be written in a simplified language.

### Understanding of the essential elements of experimental draft informed consent form

The current study found that most HCUs understood the essential elements of the informed consent form. This may have been biased by the fact that our questionnaire was administered immediately after the discussion of the informed consent information. Although there are several reports on assessment of understanding in clinical trials in Africa ^24^, there are no existing guidelines on assessment of comprehension of informed consent information used in biobanks, clinical trials and/or research studies.

Regulations and National guidelines ^5, 15^ have set forth topics that should be covered in consent forms however, there is lack of guidance on the action that should be done when a prospective participant fails to understand the information.

## Conclusions

Our study showed high HCUs acceptance of broad consent for storage of biological materials for future unspecified research uses. This was statistically significant in HCUs with a history of participating in research and those that worked in a health-related field. Almost half of the participants believed that their biological materials were destroyed immediately after the laboratory test. A shortened Informed consent form was developed and assessed by HCUs in a public health setting. A clear majority of HCUs stated that the length of the informed consent form was appropriate. HCUs that found information either not or partly understandable were less likely to support storage of their biological material and data. Therefore, understanding of the essential elements of the informed consent form is critical as a core ethical principle. A shortened and simplified informed consent form may trigger constructive discussions between potential participants and health care workers and potentially increase research participant understanding of study related materials in biobanking. The current National Research Biobanking Guidelines should be reviewed to include validated assessment tools for comprehension of informed consent information used in biobanks.

The media of presentation that information on the informed consent form is conveyed is key as supported by the participants’ suggestions that the informed consent forms should be translated into local languages. Other innovative ways of conveying the information on the consent forms to HCUs should be used. For example Health care facilities could develop short video clips on what and how human materials are used in research and the role of the National Biorepository as a resource for scientific research. 

## Data Availability

Harvard Dataverse: Replication Data for: Health care users’ acceptance of broad consent for storage of biological materials and associated data for research purposes in Uganda https://doi.org/10.7910/DVN/GOMGRT ^25^. This project contains the following underlying data: Original dataset.csv (For the study titled “Health care users’ acceptance of broad consent for storage of biological materials and associated data for research purposes in Uganda) Harvard Dataverse: Replication Data for: Health care users’ acceptance of broad consent for storage of biological materials and associated data for research purposes in Uganda https://doi.org/10.7910/DVN/GOMGRT ^25^. This project contains the following extended data: A copy of the experimental informed consent form A copy of the questionnaire Data are available under the terms of the Creative Commons Zero "No rights reserved" data waiver (CC0 1.0 Public domain dedication).
