# Supplementary material for: Health care users’ acceptance of broad consent for storage of biological materials and associated data for research purposes in Uganda
Source: Wellcome Open Res. 2022 Oct 4;7:73. Originally published 2022 Mar 1. [Version 2] doi: 10.12688/wellcomeopenres.17633.2 (PMC10687383; doi:10.12688/wellcomeopenres.17633.2)
Supplement: Peer review history

# Reviewer report: Reviewer response for version 2

**DOI:** 10.21956/wellcomeopenres.20429.r64749

**Published:** 2023-11-29

**Authors:** Zisis Kozlakidis

**Affiliations:** 1 International Agency for Research on Cancer, World Health Organization, Lyon, France

This is a well-structured study, and the limitations are clearly indicated.

 The introduction is sufficiently detailed. It is unclear how representative the HCUs are to the rest of the Ugandan population - a succinct statement should be made by the authors to that effect.

 The discussion presents the results well within the Ugandan context, it would benefit by comparing and contrasting to the few similar, other studies that have taken place in sub-Saharan Africa on the subject of broad consent. Currently it is mentioned in passing.

 The authors correctly state: "there is lack of guidance on the action that should be done when a prospective participant fails to understand the information" - however, they provide no potential recommendation on what may be appropriate within the Ugandan context; or as the basis for future research on the topic.

Is the work clearly and accurately presented and does it cite the current literature?

Yes

If applicable, is the statistical analysis and its interpretation appropriate?

Yes

Are all the source data underlying the results available to ensure full reproducibility?

Yes

Is the study design appropriate and is the work technically sound?

Yes

Are the conclusions drawn adequately supported by the results?

Yes

Are sufficient details of methods and analysis provided to allow replication by others?

Yes

Reviewer Expertise:

Biobanking; microbiology; public health

I confirm that I have read this submission and believe that I have an appropriate level of expertise to confirm that it is of an acceptable scientific standard.

# Reviewer report: Reviewer response for version 2

**DOI:** 10.21956/wellcomeopenres.20429.r69514

**Published:** 2023-11-24

**Authors:** Mariana Dittborn

**Affiliations:** 1 Great Ormond Street Hospital for Children, London, UK

Many thanks for inviting me to review the manuscript. The article offers the results of a very interesting and relevant study on broad consent acceptability in Uganda and contributes to the wider debate around informed consent models for storage of specimens and data in biobanks. 

 Have some comments to the authors with the aim of strengthening the study’s report and contributions. 

 Please offer a definition for broad consent. 

 When discussing “unique challenges” associated with consent for Biobanking, please add examples and references. What are the benefits and limitations of this type of consent? 

 The authors present 5 objectives, which seem somehow confusing. Would it be possible to have one main aim and specific objectives? I.e., Aim: Determine healthcare users acceptability of broad consent for storage of biological material and data. And specific objectives clarifying the different aspects the study will address including understanding, attitudes, motivations and perceptions. These should be clearly defined clarifying what each of these dimensions explored. 

 Please write HCUs in full the first time it appears in the text (Pag. 3, para 3)

 Unfortunately, I don’t have the expertise to comment on statistical tests applied in this study. Suggest having an expert reviewer to comment on this area. 

 Please use consistent terminology to refer to the shortened experimental informed consent form throughout the text. It would be very helpful to include it as a Table/Box/Figure as it is essential to understanding the results. In this line, was it clarified to participants that the form was an hypothetical form? Was acceptability measured as yes/no after participants read this hypothetical from? 

 Results: 

 Background characteristics of the HCU enrolled at the selected Primary Health Care facilities. Are these characteristics of the universe population or participants? How many HCU participated? How many did not participate? 

 These statements need clarification:

 Page 4, last para. “Informed consent information that is either partly or not understandable was found to be statistically significant (OR=0.27, 95% CI [0.15-0.46]) and (OR=0.10, 95% CI [0.03-0.32]) respectively as compared to informed consent information perceived as understandable.” 

 “Difficult language was found to be statistically significant (OR= 2.65, 95% CI [1.30-5.39]).”

 Table 1. Please add the total number of participants. 

 Pag 5. First para. When saying that most participants incorrectly believed that their specimens and data would only be used by the National Biorepository - was this information included in the hypothetical Informed Consent form? Please clarify. 

 Table 2. Please add the total number of respondents for reference. Are percentages taken from a total of 431 or total respondents? 

 Page 6, last para. Reasons given for why HCU wanted feedback. Were these obtained via open ended questions? Please clarify. 

 Table 3. Please add the total number of participants. Please add total numbers for each answer, as well as the numbers for “accept” and “decline” and clarify what total number the percentages refer to. 

 Some statements are somehow confusing as they use double negatives. I.e., “Did not understand the technical terms” Yes 79, does that mean that 79 participants did not understand the technical terms? Please clarify. 

 Similarly, “not interested in the content”. Please clarify. 

 Not sure about what Figure 1 adds, as it is the same data shown in Table 3. Please clarify/justify. 

 Table 4. Please add the total number of participants. Please mention which is the correct answer for each question.  

 Table 5. Please add the total number of participants. 

 Figure 2. Please add the total number of participants. Could participants select more than one answer for this question? Please clarify. 

 “HCUs with a history of participating in health-related research were statistically different from those that had never participated (p-value = 0.019). And HCUs with a history of professional work in a medical field were statistically different from those that worked in non-medical fields (p-value = 0.005).” Please clarify how were these groups statistically different. 

 “Compared to participants who perceived that the informed consent information is understandable (OR=0.10, 95% CI [0.03-0.32], participants who either partly or totally disagreed were significantly less likely to perceive information as understandable (OR=0.27, 95% CI [0.15-0.46]. “ Partially or totally disagree with? I cannot see any questions that include partially or totally disagreement (like Likert scales?). Please clarify. 

 Suggest moving the limitations to the discussion section, and explain how the exclusion of non-English fluent HCUs might have affected the results and its generalizability. 

 Please describe LMICs in full the first time it appears in the text (Discussion section, second para). 

 Pag 11, second para “HCUs responses on how to improve the broad consent form were articulated as follows: (a) Simplify the technical terms and language used. (b) The Informed consent form should be translated in various languages. (c) Shorten the informed consent form to make it brief. (d) Offer participants enough time to understand the information. (e) More sensitization of the informed consent process should be done. (f) The examples for possible re-use of biological materials should be written in a simplified language.” This should go in the results section. 

 The discussion could be strengthened by adding references to existing literature and recommendations for further research and practice that can be drawn from this study. 

 Hope these comments are helpful. I would be happy to review an updated version.

Is the work clearly and accurately presented and does it cite the current literature?

Partly

If applicable, is the statistical analysis and its interpretation appropriate?

I cannot comment. A qualified statistician is required.

Are all the source data underlying the results available to ensure full reproducibility?

Partly

Is the study design appropriate and is the work technically sound?

Partly

Are the conclusions drawn adequately supported by the results?

Yes

Are sufficient details of methods and analysis provided to allow replication by others?

Partly

Reviewer Expertise:

Clinical ethics, research ethics, end of life and palliative care, paediatrics.

I confirm that I have read this submission and believe that I have an appropriate level of expertise to confirm that it is of an acceptable scientific standard, however I have significant reservations, as outlined above.

# Reviewer report: Reviewer response for version 1

**DOI:** 10.21956/wellcomeopenres.19505.r48966

**Published:** 2022-05-05

**Authors:** Reinder Broekstra

**Affiliations:** 1 Health Psychology Research, Health Sciences, Faculty of Medicine, University of Groningen, Groningen, The Netherlands; 2 Clinical Ethics and Law Southampton (CELS), Faculty of Medicine, University of Southampton, Southampton, UK

**Review Health care users’ acceptance of broad consent for storage of biological materials and associated data for research purposes in Uganda**

 This manuscript reports on an interesting and truly relevant study on information and hypothetical consent procedure for a biorepository in Uganda. Additionally, authors apply an open science approach, which deserves compliments. However, before indexing the manuscript requires major revision. 

**General issues**

 Overall, there is a lack of transparency or clarity in the report of this study. It is not possible yet to understand or replicate the study easily. The structure of this manuscript requires improvement. Results are incorrectly reported which worries the reviewer a bit. Lastly the report is superficial in relating to the literature, description of the methods, and discussing the implications of the results.  

**Detailed comments**

 Abstract 

- “short informed consent statement (ICF)” = shortened informed consent form (ICF) or shortened statement of informed consent (IC). 
- “shortened experimental draft ICF” revise to shortened or experimental, since the draft is used eventually. 

Background 

- Bio-repository = biorepository 

- Bio-banking = biobanking 
- “Consent for bio-banking for specified or unspecified future research raises unique challenges and concerns for informed decision making especially in the context of clinical care.”:  Explain, give some examples, and add references. 
- “Broad consent provides participants with a choice about whether to allow their stored specimens to be used in future research based on a broad category, e.g., cancer, heart disease, or behavioural research ( 9).”:  Better define broad consent and relate to the literature, since there much more literature discussing consent and the complexity of being informed. For example, Manson, 2019 ^1^. Or Horton R, Lucassen A., 2019 ^2^. 
- "However, a few researchers argue that broad consent may provide participants with insufficient information to make a reasonable choice ( 10) while others may change their minds in the future ( 11).” : this is somewhat an understatement of the ongoing debate on consent in literature. Additionally, several studies show the lack of understanding of consent and preferences about broad consent, for example Ballard *et al., 2020[re-3]. *Few studies have investigated the African population, so this research conducted is very relevant and needed.  
- “Implementation of appropriate informed consent has become a cornerstone for the collection and use of biological materials and associated data from clinical care 10.” :  This is a very strange reference for this statement. 

- “Findings from this study may hopefully contribute to current debate regarding understanding the content of informed consent documents, the purpose of biobanks generally, or the scope of the future research and projects to be carried out 11.” : Why does your hope need a reference? That does not seem right. 
- “aim” = “objective”, the current descriptions of aim/objective/research question are rather confusing or not correct and overlap. Keep a revised objective and loose the rest. 

Methods 

- The study design (experimental versus non-experimental) should be written more clearly. How was the study introduced to potential participants in the study consent form, and how were research assistants trained? This might influence your outcome measure, for example being a hypothetical willingness or closer to a ‘real’ willingness. 
- Lack of description of variables chosen and rationale for the choice. It requires the reader to check the attachments to get some understanding. The authors should help the reader by providing more information in the text.  

Results 

- Table 2, percentages do not correspond to the relevant information in table 1 and table 2. It would help to have a comparison per characteristic of accepted versus declined. 
- Prevalence is a strange term to use in this context. 
- It would be good to understand how motivations are associated with other characteristics, for example perform some anova or t-tests or multiple logistic regression. Do older people have different motivations than younger people? Is there an association between educational level and motivation? 
- “HCUs with a history of participating in health-related research were statistically different from those that had never participated (p-value = 0.019). And HCUs with a history of professional work in a medical field were statistically different from those that worked in non-medical fields (p-value = 0.005).” How were these different? 
- Table 4. Show the reader what the correct answers were because some do not know or do not have time to check your documents themselves. 

Discussion 

- General lack of comparing the results of this study with other studies in literature, and discussing the ethical and research implications. 
- The following reads as results, but is mentioned as discussion. Is this correct? 
  “Almost half of the participants (43.8%) believed that remnant biospecimens were destroyed immediately after laboratory tests. Only 6.6 % and 21.3% thought clinical specimens might be stored for a specified period and for research purposes, respectively. This data may guide in the development of Standard Operating Procedures for sample retention times for biological materials used for clinical purposes that have not been consented for storage. This suggests that sample storage and further use possibilities should be more clearly specified at the time of sampling.
**Perception of format of experimental draft informed consent form **
  A shortened draft experimental Informed consent form was developed and tested with HCUs in a public health setting. One of the objectives of this study was to determine HCUs’ perception of the length and technical content of the informed consent form. Our data suggest that 82.3% of HCUs found that the length of the draft informed consent form was appropriate. Sixty five percent of HCUs who accepted storage of biological materials and data indicated that they did not understand some technical terms. However, acceptability of storage of biological materials was not a statistically significant preference. HCUs who could not understand some of the draft ICF were 90% less likely to accept storage of their biological material and data for research purposes. Those who partly understood the draft ICF were 73% less likely to accept storage of their biological material and data for research purposes. Lengthy informed consent forms remain common in research settings in LMICs, despite the fact that they negatively affect potential participants’ understanding about the proposed research. A shortened informed consent form may prompt better understanding and willingness to consent, as has been shown elsewhere 18, 19.” 

Is the work clearly and accurately presented and does it cite the current literature?

Partly

If applicable, is the statistical analysis and its interpretation appropriate?

Partly

Are all the source data underlying the results available to ensure full reproducibility?

Yes

Is the study design appropriate and is the work technically sound?

Partly

Are the conclusions drawn adequately supported by the results?

Partly

Are sufficient details of methods and analysis provided to allow replication by others?

No

Reviewer Expertise:

Ethical, Legal, and Social Aspects of innovative (Information) Technology in biomedical sciences and Medicine.

I confirm that I have read this submission and believe that I have an appropriate level of expertise to confirm that it is of an acceptable scientific standard, however I have significant reservations, as outlined above.

## References

[1] : The ethics of biobanking: Assessing the right to control problem for broad consent. *Bioethics* .33(5) : 10.1111/bioe.12550 540-549 10.1111/bioe.12550 30667086

[2] : Consent and Autonomy in the Genomics Era. *Curr Genet Med Rep* .2019;7(2) : 10.1007/s40142-019-00164-9 85-91 10.1007/s40142-019-00164-9 31281738PMC6584705

[3] : Exploring broad consent in the context of the 100,000 Genomes Project: a mixed methods study. *Eur J Hum Genet* .28(6) : 10.1038/s41431-019-0570-7 732-741 10.1038/s41431-019-0570-7 31919452PMC7253456

# Response

**Published:** 2022-09-27

**Authors:** Hellen Nansumba

**Affiliations:** Central Public Health Laboratories, Uganda

This is a cross sectional study design that was performed in 3 Health Facilities. A shortened experimental Informed Consent Form (ICF) was used.

# Reviewer report: Reviewer response for version 1

**DOI:** 10.21956/wellcomeopenres.19505.r48967

**Published:** 2022-03-25

**Authors:** Paulina Tindana

**Affiliations:** 1 Health Policy, Plannng and Management, School of Public Health, College of Health Sciences, University of Ghana School of Public Health, Accra, Ghana

This paper addresses an important ethical issue in research involving human biological materials and associated data. It reports on the findings of a cross sectional study which assessed healthcare users' acceptance of Broad Consent for the collection, storage and future use of human biological materials in Uganda 

Some comments for consideration

 The study design is sound and appropriate for addressing the research aims and objectives, but the presentation of the results and narrative needs to be improved. 

 Authors’ presentation and interpretation of participants perceptions derived from logistic regression analysis are not accurate. These results as presented do not highlight the comparison group for proper interpretation. e.g. *"informed consent information that is either partly or not understandable was...significant (OR=0.27, CI [0.15-0.46]) & (OR=0.10, CI[0.03-0.32])".* This should be revised to read along the lines of, "compared to participants who perceived that the informed consent information is understandable, participants who either partly or totally disagreed were significantly less likely to perceive the information as understandable" *.* Please revise these sections to convey the right interpretation of the regression tables. 

 ORs from regression analysis should be adjusted for confounders e.g level of education attained, others.

**Results:**

 The use of large to describe proportions estimated as percentages should be revised. The convention is either 'high' or 'low'. Please revise such statement as, *"The largest group (49.40%)"* to read "A high percentage (or proportion if you like) of HCUs had..." - Please revise all such statements accordingly .

 Data presentation should be consistent. Please specify 95% CI throughout the results. Not included in the summary description of table 3.

 Based on the authors’ statement that *"there are no existing guidelines on assessment of comprehension of informed consent information used in biobanks, clinical trials and/or research studies’  - *are there any suggestions for improving the current Ugandan National Research Biobanking Guidelines?

 The authors' suggestion that a shortened consent form will enhance understanding over looks other data driven suggestions for enhancing the general understanding of elements of consent. How the information on the consent form is conveyed is key as supported by the participants' suggestions that the form should be translated into local languages. The authors could recommend other innovative ways of conveying the information on the consent forms to healthcare users. For example, healthcare facilities could develop short video clips on what and how human biological materials are used in research and the role of the National Bio-repository as a resource for scientific research.

 Overall, this is a very good paper and will be of interest to the broader readership. The authors conclude that acceptability of Broad Consent was high among healthcare users and that participants are more likely to accept this model of consent if they understand the purpose of future uses of human biological samples. The recommendations drawn from the study are important and could inform other research initiatives on how understanding of research could be enhanced to support the ethical use of biological samples and associated data.

 Under *Acknowledgement*, with more than one author listed, all the singular pronouns should be changed to plural i.e. ‘ *we* would like to thank *my* participants’ should be ‘ *our *participants’ etc.

Is the work clearly and accurately presented and does it cite the current literature?

Yes

If applicable, is the statistical analysis and its interpretation appropriate?

Partly

Are all the source data underlying the results available to ensure full reproducibility?

Yes

Is the study design appropriate and is the work technically sound?

Yes

Are the conclusions drawn adequately supported by the results?

Yes

Are sufficient details of methods and analysis provided to allow replication by others?

Yes

Reviewer Expertise:

Public health, bioethics, social science research, research ethics, health policy

I confirm that I have read this submission and believe that I have an appropriate level of expertise to confirm that it is of an acceptable scientific standard, however I have significant reservations, as outlined above.

# Response

**Published:** 2022-09-26

**Authors:** Hellen Nansumba

**Affiliations:** Central Public Health Laboratories, Uganda

1. One of the limitations is that this study did not adjust for key confounding variables such as level of education. However, Health care users' that are illiterate of the English language were excluded from the study since the draft Experimental informed consent form about Biobanking was written in English language
2. The National Research Biobanking Guidelines should be reviewed to include a validated assessment tool for comprehension of ICFs used in Biobanking
3. The media of presentation of the information on the ICFs is conveyed is key as supported by the participants' suggestions that the Informed Consent form should be translated into local languages
4. Other innovative ways of conveying the information on the ICFs to HCUs should be adopted. For example development of short video clips on what and how human biological materials are used in research and the role of the National Biorepository as a resource for scientific research
